# Epidemiology of asylum seekers and refugees at the Mexico-US border: a cross-sectional analysis from the migrant settlement camp in Matamoros, Mexico

**DOI:** 10.1186/s12889-024-17947-7

**Published:** 2024-02-16

**Authors:** Christopher W. Reynolds, Allison W. Cheung, Sarah Draugelis, Samuel Bishop, Amir M. Mohareb, Ernesto Miguel Merino Almaguer, Yadira Benitez López, Lestter Enjamio Guerra, Raymond Rosenbloom, Joanna Hua, Callie VanWinkle, Pratik Vadlamudi, Vikas Kotagal, Florian Schmitzberger

**Affiliations:** 1 University of Michigan Medical School, 1301 Catherine St, Ann Arbor, MI 48109 USA; 2 Global Response Medicine, 7959 N Thornydale Rd, Tucson, AZ 85741 USA; 3 Team fEMR, 25615 Jefferson Ave, St. Clair Shores, MI 48081 USA; 4 Center for Global Health, Massachusetts General Hospital, 125 Nashua Street, Suite 722, Boston, MA 02138 USA; 5 Harvard Medical School, 25 Shattuck St, Boston, MA 02115 USA; 6 Division of Infectious Diseases, Massachusetts General Hospital, 55 Fruit St., Boston, MA 02114 USA; 7 Medical School for International Health, Faculty of Health Sciences, Ben-Gurion University of the Negev, 8410501 Beer-Sheva, Israel; 8 Department of Neurology, University of Michigan, 1500 E. Medical Center Drive, Ann Arbor, MI 48109 USA; 9 Ann Arbor Veterans Affairs Healthcare System (VAAAHS) and GRECC, 2215 Fuller Rd, Ann Arbor, MI 48105 USA; 10 Department of Emergency Medicine, University of Michigan, 1500 E. Medical Center Drive, Ann Arbor, MI 48109 USA

**Keywords:** Human migration, Asylum seekers, Refugees, Global health, Emigration and immigration, Refugee camps, Relief work

## Abstract

**Background:**

The number of migrants and asylum seekers at the Mexico-US border has increased to historic levels. Our objective was to determine the medical diagnoses and treatments of migrating people seeking care in humanitarian clinics in Matamoros, Mexico.

**Methods:**

We conducted a cross-sectional study of patient encounters by migrating people through a humanitarian clinic in Matamoros, Mexico, from November 22, 2019, to March 18, 2021. The clinics were operated by Global Response Medicine in concert with local non-governmental organizations. Clinical encounters were each coded to the appropriate ICD-10/CPT code and categorized according to organ system. We categorized medications using the WHO List of Essential Medicines and used multivariable logistic regression to determine associations between demographic variables and condition frequency.

**Results:**

We found a total of 8,156 clinical encounters, which included 9,744 diagnoses encompassing 132 conditions (median age 26.8 years, female sex 58.2%). People originated from 24 countries, with the majority from Central America (*n* = 5598, 68.6%). The most common conditions were respiratory (*n* = 1466, 15.0%), musculoskeletal (*n* = 1081, 11.1%), and skin diseases (*n* = 473, 4.8%). Children were at higher risk for respiratory disease (aOR = 1.84, 95% CI: 1.61–2.10), while older adults had greater risk for joint disorders (aOR = 3.35, 95% CI: 1.73–6.02). Women had decreased risk for injury (aOR = 0.50, 95% CI: 0.40–0.63) and higher risk for genitourinary diseases (aOR = 4.99, 95% CI: 3.72–6.85) compared with men. Among 10,405 medications administered, analgesics were the most common (*n* = 3190, 30.7%) followed by anti-infectives (*n* = 2175, 21.1%).

**Conclusions:**

In this large study of a migrating population at the Mexico-US border, we found a variety of clinical conditions, with respiratory, musculoskeletal, and skin illnesses the most common in this study period which encompassed a period of restrictive immigration policy and the first year of the COVID-19 pandemic.

**Supplementary Information:**

The online version contains supplementary material available at 10.1186/s12889-024-17947-7.

## Background

Over the past five years, there has been a record number of migrants, refugees, and asylum seekers seeking entry into the US at the Mexico-US border. Recent estimates by US Customs and Border Patrol (CBP) report more than 200,000 immigration encounters per month [1]. Beginning in 2019, changes in use of US immigration policy including the Migrant Protection Protocols (MPP) and Title 42 forced asylum seekers to remain in Mexico for their asylum claims to be processed. MPP allowed US CBP agents to deport or deny US entry of asylum seekers during the duration of their immigration proceedings [2]. This policy was discriminately applied primarily at ports of entry along the Mexico-US border, thereby especially affecting Spanish-speaking asylum seekers from Mexico, Central America, and South America [3]. Title 42, implemented by the Centers for Disease Control and Prevention (CDC) due to the COVID-19 pandemic in March 2020, further restricted travel between the US and Mexico by limiting nonessential border crossing, including of asylum seekers [4]. A growing number of people seeking entry to the US from the Mexico-US border were forced to remain in Mexico, many of whom sought shelter near US ports of entry. For example, in the Rio Grande Valley, in the Southeast border between Texas, USA, and Tamaulipas, Mexico, thousands of migrating people have been living in informal encampments in the border city of Matamoros, Mexico [5]. Consequently, more than 70,000 adults and children have lived in these encampments, facing increased susceptibility to diseases, psychological distress such as post-traumatic stress disorder (PTSD) and depression, COVID-19 infection, violence, and limited access to health services and the social determinants of health [5–8].

Humanitarian aid non-governmental organizations (NGOs) serve as the primary health service providers for migrants at the Mexico-US border, with limited options for tertiary and emergency care [9]. For example, in the migrant camp in Matamoros, Mexico, healthcare administration was supervised by Mexico’s *Instituto Nacional de Migración*, but delivered by NGOs including Global Response Medicine (GRM) and Médicos Sin Fronteras (MSF) [6]. Conditions in these settlements frequently lack important public health infrastructure, including water, sanitation, and hygiene (WASH) measures, adequate shelter, potable water, and safety [6]. As a result, GRM anticipated significant health challenges at the encampment, including respiratory disease, gastrointestinal illnesses, and limited resources to care for patients including insufficient diagnostic equipment, therapeutic modalities, and infrastructure for continuity of care [6, 9]. The association between persons seeking asylum and heightened vulnerability to poor health has been well documented [10, 11], and access to medical services while in transit and within camps is limited for these populations [12].

Despite asylum seekers’ vulnerability to poor health outcomes and access, there is a dearth of information on asylum seekers' health conditions while waiting at the Mexico-US border [13]. This crucial knowledge gap of asylum seekers’ disease burden and effective health delivery models for US-bound migrants is due in part to the lack of data recording mechanisms for this population. While a few studies have assessed the mental health status of asylum seekers at the Mexico-US border, quantitative studies of medical illnesses with large scale populations have not been previously described. We sought to address this gap by assessing the disease burden of asylum seekers living in a tent encampment in Matamoros, Mexico. Specifically, we analyzed patient characteristics, frequency of diagnostic codes, association of age and sex with diagnoses, and medications administered.

## Methods

### Study design and population

We conducted a cross-sectional study of electronic medical data from patient encounters in Matamoros, Mexico from November 22, 2019, to March 18, 2021. This study period represented the entire time of operation that GRM provided medical care in the Matamoros camp, with the first clinical encounter occurring in November 22, 2019, and continuing until GRM’s suspension of services in March 2021 due to closure of the camp. Patients included asylum seekers, refugees, and other migrating people who presented for medical care in either of two clinics operated by GRM in an informal encampment in Matamoros, Mexico. No formal census of the population of migrating people in this city is available, but internal estimates of population size ranged between 1,000 and 3,500 residents with nearly 500 children, depending on time periods, with a high turnover in population [6]. Due to MPP and Title 42, the population waited anywhere from months to two years to enter the United States. GRM delivered services at two locations: a mobile health trailer clinic within the asylum seeker camp, and a two-story clinic in a permanent building directly across from the camp, with three private consultation rooms and access to basic imaging including ultrasound and X-ray. The clinic provided medical care that was free at the point of access without discrimination based on country of origin, immigration status, or ability to pay. The clinic was staffed by local and volunteer clinicians from either Mexico or the US. Clinic staff also included volunteer healthcare workers from the migrating and asylum seeker population who had medical training in their home countries or who were able to provide interpreting services. Given resource limitations, particularly with availability of laboratory and diagnostic testing, most diagnoses were based on clinical signs, symptoms, and physical exam findings. For example, when evaluating patients for COVID-19, clinical symptoms and exposures often formed the basis of a diagnosis. Diagnostic testing with antibody and antigen tests were made available later during the study period, Medications were distributed to patients at the clinical site, and patients requiring specialty or hospital referral were provided with assistance in accessing local specialists or acute medical services in the area. Médicos Sin Fronteras (MSF) was also present in the camp, offering primarily mental health services with occasional support for physical medical conditions among the population. Patients seeking those services would have presented directly to MSF to be evaluated and otherwise would have been advised to do so by GRM providers.

### Patient and public involvement

An initiative from GRM and Team fEMR (Fast Electronic Medical Record), this work represents a collaboration including local humanitarian workers, researchers with volunteer experience at the Mexico-US border, and asylum seekers who volunteered as medical personnel to work with GRM as clinicians and interpreters. Patients were not involved in the design or conduct of this study.

### Data sources

We used the electronic medical data for all patient encounters in GRM’s Matamoros clinic operations from November 22, 2019, to March 18, 2021. Clinicians documented all clinical encounters using Fast Electronic Medical Record (fEMR), an electronic medical record system specifically designed for use in humanitarian response and resource-limited settings [14]. fEMR is programmed to optimize usability, characterize diagnoses and treatments of patients, and provide access to a medical record for people in these settings [15]. The system has an easily usable interface and requires minimal on-boarding. All patients were registered into fEMR with a unique medical reference number using government-issued identification, which could include a passport from one’s home country or migration document provided by the government of Mexico. Patients reported their age, sex assigned at birth, and country of origin. When patients returned for clinical visits, they were frequently, but not uniformly, registered under their prior medical reference number. Recording patients with unique medical identifiers served as a control mechanism to avoid repeat patients There is no minimal amount of clinical documentation required to complete a patient encounter, so variation in the amount of clinical information available for review did exist between clinical visits.

To standardize the records, we had two trained clinical coders independently review each clinical encounter and assign International Classification of Diseases, Tenth Revision, Clinical Modification (ICD-10-CM), Current Procedural Terminology (CPT), and Healthcare Common Procedure Coding System (HCPCS) codes for each encounter for the entire dataset. A third, experienced coder reviewed the codes assigned to each encounter for consistency and arbitrated the coding in cases of discrepancy. Patient encounters could receive more than one diagnosis from one clinical encounter. All coders were subject to a qualification exam on which they had to earn higher than 70% prior to participating in the project. A total of 41 medical coders and 10 expert arbitrators worked on the current dataset. Coders were subject to onboarding training as well as HIPAA training before they could begin. Coders made no assumptions or interpretations of the medical record, and they were also unable to contact providers given the time lapse between the time of the patient encounter and the time of the record review. The final dataset, which included age, sex assigned at birth, country of origin, ICD-10, CPT/HCPCS codes, and a list of dispensed medications, were completely stripped of all identifiers.

### Outcomes and covariates

We categorized diagnoses by the following organ systems or etiologies according to their ICD-10-CM classification: blood and immune system, congenital malformations, circulatory system, ear and mastoid process, endocrine system, eye and adnexa, digestive system, infectious and parasitic diseases, injury, poisoning and other external causes, genitourinary system, musculoskeletal and connective tissue, mental and neurodevelopmental disorders, neoplasms, nervous system, pregnancy, respiratory system, skin and subcutaneous tissue, and symptoms, signs, and abnormal clinical and laboratory findings not elsewhere classified [16]. Since the population size in the Matamoros encampment was highly variable and never precisely known, we report diagnosis codes as a frequency of total ICD-10 codes rather than prevalence. We did not specify repeat clinical encounters per patient or multiple diagnoses per encounter but analyzed each as an independent ICD-10 code. We categorized medications according to the World Health Organization’s Model List of Essential Medicines [17]. We classified countries of origin by regions including Central America, North America, Caribbean, South America, and Other. Our primary outcome was the proportion of encounters with each category of medical condition. Our covariates included age, sex assigned at birth, and country of origin.

### Statistical analysis

We report continuous variables using median and interquartile range and categorical variables using number and proportions. We used multivariable logistic regression to determine associations between diagnosis categories and age, sex, and country of origin. Of the individuals whose country of origin was in North America, only individuals originating from Mexico were included in the logistic regression due to the low sample sizes of patients originating from other North American countries. Similarly, individuals whose country of origin was located in other regions such as Africa or Oceania, were excluded from the logistic regression due to their low sample sizes. Because of the overall large sample size, we used Wald intervals to estimate the binomial 95% confidence intervals. All analyses were conducted in R (version 4.3.1), and figures were created using the *ggplot2* and *forestploter* packages [18, 19]. A map of patient country and region of origin was created in Microsoft Excel. In order to preserve the anonymity of our patient population, countries with fewer than 20 patients were grouped by region and not reported directly on the country of origin figure.

### Ethical compliance

This study was granted “not regulated” status by the Institutional Review Board at University of Michigan Medical School (HUM00222340) and conformed to the principles embodied in the Declaration of Helsinki. Informed consent was obtained by all patients prior to receiving medical evaluation. For the purposes of this retrospective study, informed consent was not obtained by subjects as all medical information was de-identified and therefore impossible to identify participants to undergo the consent process.

## Results

### Demographics

**Fig. 1:**
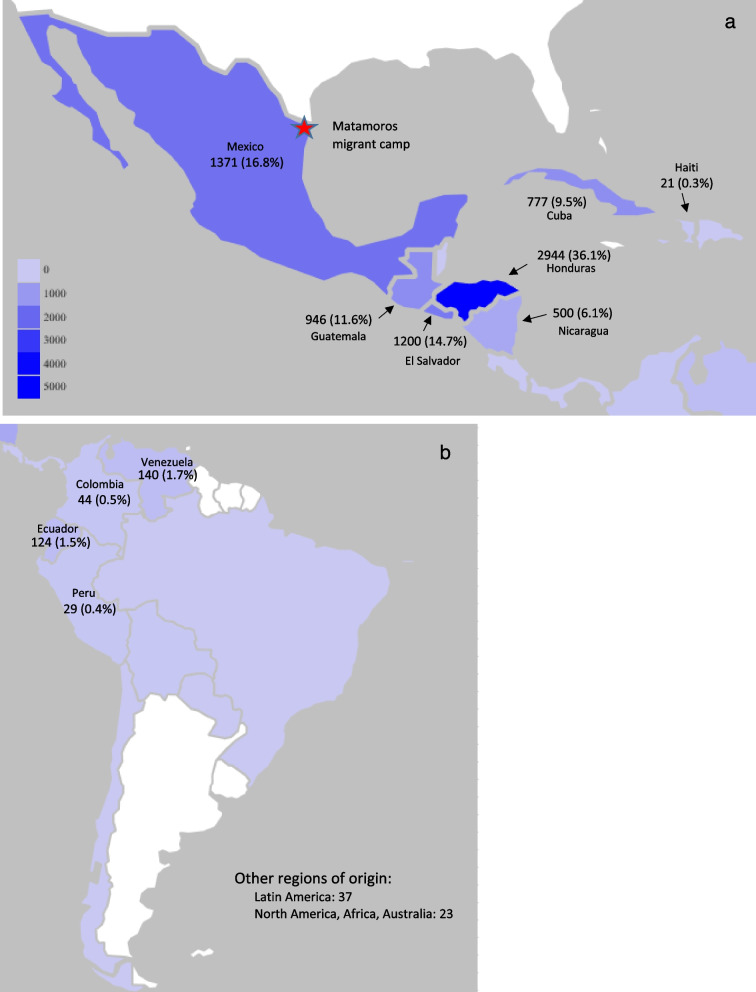
**a** and **b** Map of Self-reported Country of Origin of Asylum Seekers utilizing Health Services in Matamoros, Mexico encampment from November 2019 to March 2021. Legend: Self-reported Countries of origin of asylum seekers in Matamoros, Mexico, 2019–2021 from Mexico and Central (1a) and South (1b) America. Countries with fewer than 20 patients were aggregated into the “Other” category to guarantee anonymity and not reported directly on Fig. 1. Of note, these are self-reported countries of origin which were recorded by clinicians as part of the clinical encounter. There may be limitations due to these being self-reported. These include migrants who have been traveling for years reporting their most recent country of emigration (as is common among Haitian migrants traveling from Brazil and Chile), or the most recent country of visitation (such as the United States and Canada). However, those identifying as being from the United States likely were but were deported at a young age

Over the study period of 483 days (1 year, 3 months, 24 days), 8,156 patient encounters were recorded by the GRM Matamoros clinics (Table 1). These encounters occurred among 2,876 unique medical identifiers which indicated an approximate total patient number. People were mostly female (*n* = 4,748, 58.2%) and median (IQR) age was 26.8 (8.0 – 37.5) years. There were 24 different countries of origin represented in the dataset with the majority (*n* = 5,598, 68.6%) from Central America (Fig. 1).

**Table 1:** Demographics of asylum seekers utilizing health care services in the Matamoros, Mexico camp November 2019-March 2021

Demographics	Frequency (%)
	(*N* = 8156 patient encounters)
Sex	
Female	4748 (58.2%)
Male	3408 (41.8%)
Age	
Median [Min, Max]	26.8 [0, 80.3]
Age range	
< 18	3010 (36.9%)
18–25	915 (11.2%)
26–49	3556 (43.6%)
50–64	588 (7.2%)
> 65	86 (1.1%)
Region of origin	
Central America	5598 (68.6%)
Honduras	2944 (36.1%)
El Salvador	1200 (14.7%)
Guatemala	946 (11.6%)
Nicaragua	500 (6.1%)
Other	8 (< 0.1%)
North America	1392 (17.1%)
Mexico	1371 (16.8%)
USA or Canada	21 (0.3%)
Caribbean	798 (9.8%)
Cuba	777 (9.5%)
Haiti	21 (0.3%)
South America	366 (4.5%)
Venezuela	140 (1.7%)
Ecuador	124 (1.5%)
Colombia	44 (0.5%)
Other	58 (0.7%)
Other regions	2 (< 0.1%)

### Frequency of diagnostic codes

Among 8,156 clinical encounters, a total of 9,744 diagnoses encompassing 132 unique conditions were made for migrant patients (Table 2). Diagnoses affected all organ systems, including the respiratory system; musculoskeletal system and connective tissue; skin and subcutaneous tissue; genitourinary system; digestive system; eye and adnexa; ear and mastoid process; endocrine, nutritional, and metabolic systems; circulatory system; nervous system; and blood and blood-forming organs (Supplemental Material 1). Diseases not otherwise classified by organ system fell into etiologic ICD-10 coding categories including certain infectious and parasitic diseases; diseases due to injury, poisoning, or other external causes; congenital malformations; neoplasms; mental and behavioral disorders; and diagnoses associated with pregnancy, childbirth, or the peripartum period. The most common ICD-10 diagnosis categories included diseases of the respiratory system (J00-J99, *n* = 1,466 (15.0% of patient encounters)), diseases of the musculoskeletal system and connective tissue (M00-M99, *n* = 1,081 (11.1%)), and diseases of the skin and subcutaneous tissue (L00-L99, *n* = 473 (4.8%)). Infectious and parasitic diseases comprised approximately 2% of all diagnoses, with the most common being mycoses (*n* = 78, 0.8%).

**Table 2:** Frequency of diagnostic codes according to organ system by ICD-10 classification among asylum seekers in the Matamoros, Mexico encampment, 2019–2021

ICD-10 classification	ICD-10 Sub-Chapter	Frequency (%)
		(*N* = 9744 diagnoses)
Symptoms, signs and abnormal clinical and laboratory findings, not elsewhere classified		4101 (42%)
Diseases of the respiratory system		1466 (15.0%)
	Acute Upper Respiratory Infections	719 (7.4%)
	Influenza And Pneumonia	430 (4.4%)
	Other Diseases Of Upper Respiratory Tract	267 (2.7%)
Diseases of the musculoskeletal system and connective tissue		1081 (11.1%)
	Other Dorsopathies	440 (4.5%)
	Other Soft Tissue Disorders	340 (3.5%)
	Other Joint Disorders	262 (2.7%)
Diseases of the skin and subcutaneous tissue		473 (4.8%)
	Dermatitis And Eczema	221 (2.3%)
	Other Disorders Of The Skin And Subcutaneous Tissue	120 (1.2%)
	Disorders Of Skin Appendages	60 (0.6%)
	Infections Of the Skin And Subcutaneous Tissue	42 (0.4%)
Diseases of the genitourinary system		451 (4.6%)
	Noninflammatory Disorders Of Female Genital Tract	306 (3.1%)
	Disorders Of Breast	49 (0.5%)
	Inflammatory Diseases Of Female Pelvic Organs	31 (0.3%)
Diseases of the digestive system		435 (4.5%)
	Diseases Of Oral Cavity And Salivary Glands	270 (2.8%)
	Other Diseases Of Intestines	68 (0.7%)
	Diseases Of Esophagus, Stomach And Duodenum	65 (0.7%)
Injury, poisoning and certain other consequences of external causes		346 (3.5%)
	Injuries To The Head	75 (0.8%)
	Injuries To The Ankle And Foot	49 (0.5%)
	Other And Unspecified Effects Of External Causes	39 (0.4%)
Diseases of the eye and adnexa		311 (3.2%)
	Other Disorders Of Eye And Adnexa	179 (1.8%)
	Disorders Of Eyelid, Lacrimal System And Orbit	50 (0.5%)
	Visual Disturbances And Blindness	39 (0.4%)
Diseases of the ear and mastoid process		293 (3%)
	Other Disorders Of Ear	264 (2.7%)
	Diseases Of External Ear	13 (0.1%)
	Diseases Of Middle Ear And Mastoid	11 (0.1%)
Certain infectious and parasitic diseases		176 (1.8%)
	Mycoses	78 (0.8%)
	Helminthiases	32 (0.3%)
	Viral Infections Characterized By Skin And Mucous Membrane Lesions	27 (0.3%)
	Pediculosis, Acariasis And Other Infestations	20 (0.2%)
Endocrine, nutritional and metabolic diseases		144 (1.5%)
	Other Nutritional Deficiencies	61 (0.6%)
	Diabetes Mellitus	49 (0.5%)
	Disorders Of Thyroid Gland	15 (0.2%)
Diseases of the nervous system		126 (1.3%)
	Episodic And Paroxysmal Disorders	103 (1.1%)
	Other Disorders Of The Nervous System	14 (0.1%)
	Nerve, Nerve Root And Plexus Disorders	5 (0.1%)
External causes of morbidity		97 (1%)
	Slipping, Tripping, Stumbling And Falls	38 (0.4%)
	Exposure To Inanimate Mechanical Forces	21 (0.2%)
	Exposure To Animate Mechanical Forces	16 (0.2%)
Diseases of the circulatory system		86 (0.9%)
	Hypertensive Diseases	70 (0.7%)
	Pulmonary Heart Disease And Diseases Of Pulmonary Circulation	6 (0.1%)
	Other Forms Of Heart Disease	5 (0.1%)
Pregnancy, childbirth and the puerperium		85 (0.9%)
	Other Maternal Disorders Predominantly Related To Pregnancy	46 (0.5%)
	Other Obstetric Conditions, Not Elsewhere Classified	15 (0.2%)
	Pregnancy With Abortive Outcome	14 (0.1%)
Mental, Behavioral and Neurodevelopmental disorders		45 (0.5%)
Diseases of the blood and blood-forming organs and certain disorders involving the immune mechanism		21 (0.2%)
Neoplasms		14 (0.1%)
Provisional assignment of new diseases of uncertain etiology or emergency use		12 (0.1%)
Congenital malformations, deformations and chromosomal abnormalities		11 (0.1%)

Medical diagnoses of asylum seeker patients in Matamoros, Mexico from November 2019 to March 2021, according to ICD-10 code classifications. “Diseases of the digestive system” does not include infectious gastrointestinal diseases, which are instead included under “Certain Infectious and Parasitic Diseases.” Only the most common ICD-10 subchapters are included in this table but a comprehensive list can be accessed through Supplemental Material 1

Approximately half of patient encounters (*n* = 4,101, 50.3%) received a diagnosis of symptoms, signs, and abnormal findings that were “not elsewhere classified” (also known as R00-R99 codes, or “R code”). More than one-third of patients received only an R code (*n* = 3,107, 38.1% of patient encounters), while the remaining received an R code with one or more other diagnoses. Of those receiving only R codes, the most common reported symptoms and signs were abdominal pain (*n* = 979, 31.5%), fever (*n* = 740, 23.8%), cough (*n* = 723, 23.3%), headache (*n* = 518, 16.7%), and diarrhea (*n* = 423, 13.6%). R code frequency fluctuated during the study period from 37.5%-60% as a proportion of the overall ICD-10 count. At least 400 patient encounters were recorded to have more complex pathologies requiring extensive workups, including neoplasms, ophthalmologic disorders, thyroid disease, heart disease, and disorders of the central nervous system.

### Patient factors associated with diagnostic code frequency

**Fig. 2:**
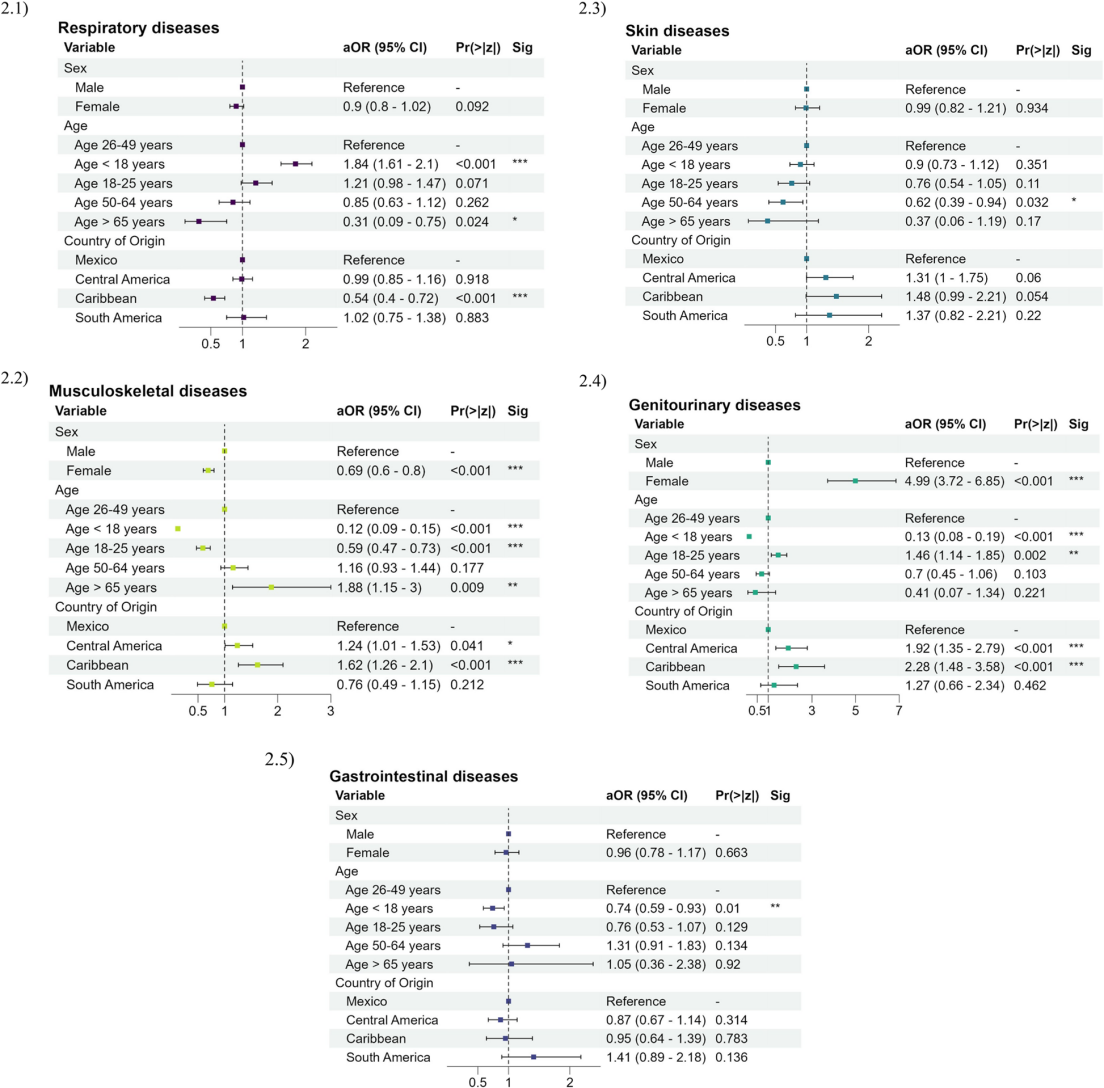
Demographic risk factors for the five most common ICD-10 code disease categories for asylum seekers in Matamoros, Mexico. Legend: Forest plot of demographic risk factors for the five most common ICD-10 diagnostic codes among our sample, including: respiratory, musculoskeletal, skin, gastrointestinal, and genitourinary illness. Demographic factors included in the model were region of origin (Mexico, Central America, South America, Caribbean), age category, and sex assigned at birth

Respiratory diseases were more likely in pediatric patients (aOR = 1.84, 95% CI: 1.61–2.10) and less likely in elderly patients (aOR = 0.31, 95% CI: 0.09–0.75) and those from Caribbean countries (aOR = 0.54, 95% CI: 0.40–0.72) (Fig. 2). When stratified by ICD-10 sub-chapters, patients < 18 years old were at increased risk for pneumonia and influenza (aOR = 3.24, 95% CI: 2.55–4.15), but not acute upper respiratory infections (aOR = 1.08, 95% CI: 0.91–1.28). Musculoskeletal diseases were less likely in female (aOR = 0.69, 95% CI: 0.60–0.80) and younger patients aged < 18 years (aOR = 0.12, 95% CI: 0.09–0.15) and 18–25 years (aOR = 0.59, 95% CI: 0.47–0.73), but more likely among older patients age > 65 years (aOR = 1.88, 95% CI: 1.15–3.0). Dorsopathies and soft tissue disorders reflected this trend with lower risk in females (aOR = 0.65, 95% CI: 0.52–0.82) and patients < 18 years (aOR = 0.21, 95% CI: 0.15–0.30). Genitourinary diseases were significantly more likely in females (aOR = 4.99, 95% CI: 3.72–6.85) and less likely in patients < 18 years (aOR = 0.13, 95% CI: 0.08–0.19), including for inflammatory disorders of the female genital tract (aOR = 0.10, 95% CI: 0.05–0.16 for patients < 18 years). Female patients were at lesser risk for injury compared to males (aOR = 0.50, 95% CI: 0.40–0.63). Adults ≥ 65 were at greater risk for joint disorders (aOR = 3.35, 95% CI: 1.73–6.02), diseases of the eye and adnexa (aOR = 3.26, 95% CI: 1.48–6.40), and circulatory disorders which included hypertension (aOR = 21.91, 95% CI: 10.27–44.36).

### Medication prescribing patterns

Seven thousand one hundred forty-five medications were dispensed among the patient encounters (Table 3, Supplemental Material 2). The most commonly prescribed medications included ibuprofen, acetaminophen (paracetamol), and other analgesics (*n* = 2,181, 30.5%). Antifungal, antiviral and antibiotic agents (*n* = 1,611, 22.5%) were the second most common and included clotrimazole (*n* = 369, 5.2%) and azithromycin (*n* = 230, 3.2%). Other commonly prescribed medications included multivitamins (*n* = 601, 8.4%) and antiallergics, most frequently loratadine (*n* = 407, 5.7%) and dextromethorphan with guaifenesin and phenylephrine (*n* = 375, 5.2%). There was less distribution of medications for mental and behavioral disorders (*n* = 3, 0.04%), disease of the central nervous system including gabapentin (*n* = 10, 0.1%), and neoplasms (*n* = 1, 0.01%). Additionally, there was limited recorded use of electrolytes through oral rehydration solution (*n* = 119, 1.7%) and inhaled bronchodilators including albuterol and salmeterol (*n* = 26, 3.6%).

**Table 3:** Medications distributed for migrating people in Matamoros, Mexico, 2019–2021 classified by the World Health Organization List of Essential Medicines

Medication category	Frequency (*N* = 7145)	Percentage (%)
Medicines for Pain and Palliative Care	2181	30.5
Anti-Infective Medicines	1611	22.5
Ear, Nose and Throat Medicines	1021	14.3
Antiallergics and Medicines Used in Anaphylaxis	605	8.5
Vitamins and Minerals	601	8.4
Medicines for Endocrine Disorders	443	6.2
Gastrointestinal Medicines	296	4.1
Solutions Correcting Water, Electrolyte and Acid–Base Disturbances	119	1.7
Cardiovascular Medicines	117	1.6
Dermatological Medicines (Topical)	105	1.5
Medicines for Reproductive Health and Perinatal Care	15	0.2
Ophthalmological Preparations	14	0.2
Anticonvulsants/Antiepileptics	10	0.1
Medicines for Mental and Behavioural Disorders	3	< 0.1
Diuretics	2	< 0.1
Antimigraine Medicines	1	< 0.1
Immunomodulators and Antineoplastics	1	< 0.1
TOTAL	7145	100

## Discussion

In this cross-sectional study of clinical encounters, we describe the clinical conditions and treatments provided for migrating people living in a large encampment at the Mexico-US border. Diseases affected all organ systems, with the most common being respiratory, musculoskeletal, and skin illnesses, followed by diseases of the gastrointestinal and genitourinary tracts. We found that respiratory illnesses comprised 15% of the diagnoses, with acute upper respiratory infections (7.4%) and pneumonia (4.4%) being the most common diagnoses. Skin conditions were also common in this population and included dermatitis, eczema, and skin infections. We also found variations in demographics among the population. Children were more commonly diagnosed with respiratory infections and older adults were more commonly diagnosed with musculoskeletal conditions and hypertension. Women were more commonly diagnosed with genitourinary illnesses, and men were more commonly diagnosed with injuries. Most of the medications prescribed in this setting appeared to be supportive treatments for pain, allergies, and skin diseases. Surprisingly, bronchodilators were rarely distributed, despite respiratory disease being the most common diagnosis and children being at increased risk. With medication distribution and clinical encounters at a nearly one-to-one ratio, this trend suggests limited clinician access to these medicines.

There are two novel aspects to our approach in this study. First, we investigated diagnoses made in a clinic that was directly embedded within a migrant encampment at a time of rapid growth in the population of migrating people along the Mexico-US border. The restrictive entry policies of MPP and Title 42 prevented nearly all asylum seekers from entering the US during this study period [3–5]. Second, we used an innovative methodology to gain insight into this population, by assigning ICD-10 codes to free text assessments made in busy outpatient clinics in large encampments led by humanitarian relief clinicians. To ensure consistency, we had each medical encounter with a free text diagnosis be coded twice to assign an ICD-10 and reviewed by an expert coder who arbitrated discrepancies in code. Given the challenges of delivering medical care in this setting, approximately one-third of the sample received an undifferentiated R-code diagnosis: “symptoms, signs, and abnormal findings that could not be otherwise classified.” Also notable was the variability of R code diagnoses over time, which could be due to multiple factors including clinician staffing or limited diagnostic equipment at the GRM clinic which prevented clinicians from making a specific diagnosis. Future work could determine if patient presentation or other system-level factors influenced the likelihood of receiving this nondescript classification.

Where specific diagnoses were made, we found that respiratory illnesses comprised 15% of the diagnoses and were more common in young children. This is consistent with studies of outpatient care in a variety of other settings, including high-income settings in the US and Europe [20–22]. These findings also highlight the opportunity for impact of vaccination programs, especially among children. As this study encompassed the first year of the COVID-19 pandemic and we were evaluating patients in a crowded encampment, we would have expected a higher proportion of clinical encounters to be related to acute respiratory infection. Several risk factors including overcrowding, poor nutrition, and social vulnerabilities would be likely to increase this population’s risk for COVID-19 and other infectious diseases [23], and it is possible that a minority of people experiencing low acuity symptoms of respiratory infections did not present to the clinic. However, we believe this is unlikely given the close connections between GRM staff and the encampment community and the routine symptom monitoring and screening that occurred during this early phase of the pandemic. We believe that these findings suggest that this migrating population did not have a specifically higher risk of importing or transmitting COVID-19 or other respiratory infections, which challenges the stated motivation for implementing Title 42 during the pandemic [24]. These findings support other studies focusing specifically on COVID-19 at the Mexico-US border, which show similar COVID-19 incidence and little to no correlation between immigrant entry and COVID-19 infection rates [25, 26].

We also found age- and sex-based differences in the diagnoses provided in this setting. Older adults were more commonly diagnosed with musculoskeletal conditions, joint diseases, and hypertension. Women were more commonly diagnosed with genitourinary conditions, and men were more commonly diagnosed with injuries. These patterns of differences are also consistent with what is seen in most outpatient settings, including those in high-income countries [27–29]. This suggests to us that the population studied has generally the expected age- and sex-specific health needs as would be seen in any general environment. The high number of skin conditions (4.8% of all diagnoses) is likely related to the risks of living in tent encampments. Notably, the prevalence of diagnosed mental health and psychiatric conditions was lower than what we would have expected for this population of people escaping trauma, violence, and civil unrest [30, 31]. This lower frequency could be attributed to GRM not providing mental health services in the camp, that we did not have access to information on services provided by the other major humanitarian organization, Médicos Sin Fronteras, who was the primary mental health provider in the encampment, and that there were likely many social factors which limited patient reporting including hesitancy to disclose information within a small community. Since GRM did not have capabilities to provide mental health services and MSF hired a full-time trained professional for mental health work, GRM’s clinicians were trained to refer patients to MSF when appropriate. However, similarly low rates of mental health diagnoses have been reported among asylum seekers in Europe at around 4% [22].

## Limitations

Our results need to be interpreted within the limitations of the study design and approach. Due to the cross-sectional nature, we cannot determine the clinical outcomes associated with care in this population, and we do not have access to inpatient and emergency medical care that were provided in hospital settings. However, the nature of our humanitarian clinic frequently made it the first medical point of contact for people living in this encampment, given limited access to Mexican hospitals [6, 12]. Second, mental health diagnoses are likely underreported in our dataset for services delivery and larger social factors. This may have limited our understanding of mental health and psychiatric conditions in this population. Third, country of origin was self-reported from patients themselves and should not be conflated with race or ethnicity. Fourth, past medical, surgical, and social histories were often not recorded among our cohort, limiting the comprehensiveness of our logistic models. While the fEMR interface allowed for recording of these data, this section was rarely completed. These gaps could be due to apprehension from asylum seekers with a history of trauma to reveal sensitive health information or a lack of reliable health records from asylum seeker patients [32]. GRM clinicians also reported intentionally under-recording certain demographic and event details surrounding sensitive clinical encounters, to guarantee anonymity and further protect patients and providers. Fifth, we were unable to assess dynamics of data over time due to limitations with the data period lasting only one full calendar year which encompassed the onset of the COVID-19 pandemic, natural disasters, and unaccounted for fluctuations in the camp population size. Similarly, the fEMR system did not record patients who left without being seen, number of volunteer clinicians, or length of the clinical encounter. These limitations make it difficult to accurately infer variation in clinic volume and how potential excessive demands on clinicians may affect diagnostic patterns, particularly for R codes. Finally, the de-identified dataset lacked mechanisms for patient tracking, so it was impossible to confirm if each clinical encounter corresponded to a distinct patient. Therefore, we calculated ICD-10 codes as a proportion of total diagnoses, rather than incidence or prevalence.

## Conclusion

This is one of the first studies analyzing the epidemiological profile of asylum seekers and migrating people living in tent encampments in Matamoros, Mexico, a US port of entry. Our sample demonstrated a varied disease profile, though one which was consistent with other clinical settings including higher risk for respiratory disease in children, genitourinary disease in females, and injury in males. The frequency of respiratory illnesses in this setting may have been related to the COVID-19 pandemic, though specific pathogen diagnoses were lacking, and there was no evidence that this population was at a higher risk of transmitting or importing COVID-19. Medical treatments provided in this humanitarian relief setting are mostly consistent with supportive care for pain, infection, and allergies, treatment for skin conditions, and empiric antibiotic treatments. Future studies should more specifically study how restrictive immigration policy, including MPP and Title 42, directly impacts health outcomes and service access of asylum seekers at the Mexico-US border.

### Supplementary Information

**Additional file 1: Supplementary Material 1.** Comprehensive list of ICD-10 diagnostic codes for people migrating in Matamoros, Mexico from November 2019 to March 2022.

**Additional file 2: Supplemental Material 2.** Complete list of medications distributed to migrating people in Matamoros, Mexico, categorized according to the World Health Organization List of Essential Medicines.

## Data Availability

The datasets used and/or analyzed during the current study are available from the corresponding author on reasonable request.

## Abbreviations

GRM: Global Response Medicine
MPP: Migrant Protection Protocols
ICD-10: International Classification of Diseases, Tenth Revision

## References

[1] U.S. Customs and Border Protection. Nationwide Encounters, 2023. https://www.cbp.gov/newsroom/stats/nationwide-encounters. Accessed: 21 August 2023.

[2] Migrant Protection Protocols. Department of Homeland Security. 24th January 2019.

[3] Baker JR, Timm A (2021). Zero-tolerance: the trump administration's human rights violations against migrants on the southern border. Drexel Law Review.

[4] Sherman-Stokes S (2021). Public health and the power to exclude: immigrant expulsions at the border. Georgetown Immigration Law J.

[5] Hampton K, Heisler M, Pompa C, Slavin A. Neither Safety nor Health: How Title 42 Expulsions Harm Health and Violate Rights, 2021. https://phr.org/our-work/resources/neither-safety-nor-health/. Accessed 10 Jul 2023.

[6] Reynolds CW, Ramanathan V, Das PJ, Schmitzberger F, Heisler M (2022). Public Health Challenges and Barriers to Health Care Access for Asylum Seekers at the U.S.-Mexico Border in Matamoros. Mexico. J Health Care Poor Underserved..

[7] Hampton K, Heisler M, Mishori R, Naples-Mitchell J, Raker E, Long R, et al. Forced into Danger: Human Rights Violations Resulting from the U.S. Migrant Protection Protocols, 2021. https://phr.org/our-work/resources/forced-into-danger/#:~:text=For%20the%20last%20two%20years,and%20international%20law%2C%20which%20prohibits. Accessed 15 Jul 2023.

[8] Reynolds CW, Ramanathan V, Lorenzana E, Das P, Sagal K, Lozada-Soto K (2021). Challenges and Effects of the COVID-19 Pandemic on Asylum Seeker Health at the U.S. -Mexico Border. Health Equity..

[9] Leiner A, Sammon M, Perry H, Dunavant S (2020). Facing COVID-19 and Refugee Camps on the U.S. Border. J Emerg Med..

[10] Gerritsen AA, Bramsen I, Devillé W, van Willigen L, Hovens J, van der Ploeg H (2006). Physical and mental health of Afghan, Iranian and Somali asylum seekers and refugees living in the Netherlands. Soc Psychiatry Psychiatr Epidemiol.

[11] Kleinert E, Müller F, Furaijat G, Hillermann N, Jablonka A, Happle C (2019). Does refugee status matter? Medical needs of newly arrived asylum seekers and resettlement refugees - a retrospective observational study of diagnoses in a primary care setting. Confl Health.

[12] Infante C, Vieitez-Martinez I, Rodríguez-Chávez C, Napoles G, Larrea-Schiavon S, Bojorquez I (2022). Access to health care for migrants along the Mexico-United States border: applying a framework to assess barriers to care in Mexico. Front Public Health.

[13] Beer J, Dorris CS, Fateen D, Mishori R (2023). What Do We Know About the Health Status of Asylum Seekers in the United States?: Identifying Research Gaps Following a Bibliometric Scoping Review of Existing Literature. J Ambul Care Manage..

[14] Team fEMR, 2023. https://www.TeamfEMR.org. Accessed 27 Aug 2023.

[15] Draugelis S, Zurek K, Brown EC, Ryan M, Chuang C (2016). Sutherland P.1.038 Electronic health records system as a catalyst for inter-institutional collaboration in international medical relief workAbstract presented as New and emerging priorities for global health. Ann Glob Health..

[16] World Health Organization. The ICD-10 classification of mental and behavioural disorders: Diagnostic criteria for research, 1993. https://apps.who.int/iris/handle/10665/37108. Accessed 10 June 2023.

[17] World Health Organization. WHO Model List of Essential Medicines - 22nd list, 2021. https://www.who.int/publications/i/item/WHO-MHP-HPS-EML-2021.02. Accessed 3 Jul 2023.

[18] Wickham H (2016). ggplot2: Elegant Graphics for Data Analysis.

[19] Dayimu A. forestploter: Create Flexible Forest Plot. R package version 1.1.0: 2021.

[20] May L, Mullins P, Pines J (2014). Demographic and treatment patterns for infections in ambulatory settings in the United States, 2006–2010. Acad Emerg Med.

[21] Gaffney A, Himmelstein DU, Woolhandler S (2023). Population-Level Trends in Asthma and COPD Emergency Department Visits and Hospitalizations Before and During the COVID-19 Pandemic in the US. Ann Allergy Asthma Immunol.

[22] Müller F, Kleinert E, Hillermann N, Simmenroth A, Hummers E, Scharff A (2021). Disease burden in a large cohort of asylum seekers and refugees in Germany. J Glob Health.

[23] Jaljaa A, Caminada S, Tosti ME, D'Angelo F, Angelozzi A, Isonne C (2022). Risk of SARS-CoV-2 infection in migrants and ethnic minorities compared with the general population in the European WHO region during the first year of the pandemic: a systematic review. BMC Public Health.

[24] Beckett AG, Viaud L, Heisler M, Mukherjee J (2022). Misusing public health as a pretext to end asylum - Title 42. N Engl J Med.

[25] Filosa JN, Botello-Mares A, Goodman-Meza D (2022). COVID-19 needs no passport: the interrelationship of the COVID-19 pandemic along the U.S.-Mexico border. BMC Public Health..

[26] Nwadiuko J, Bustamante AV (2022). Little To No Correlation found between immigrant entry And COVID-19 infection rates in the United States. Health Aff (Millwood).

[27] Oliveros E, Patel H, Kyung S, Fugar S, Goldberg A, Madan N (2020). Hypertension in older adults: Assessment, management, and challenges. Clin Cardiol.

[28] DiMaggio CJ, Avraham JB, Lee DC, Frangos S, Wall SP (2017). The Epidemiology of Emergency Department Trauma Discharges in the United States. Acad Emerg Med.

[29] Harrington RD, Hooton TM (2000). Urinary tract infection risk factors and gender. J Gend Specif Med..

[30] Sidamon-Eristoff AE, Cohodes EM, Gee DG, Pena CJ (2022). Trauma exposure and mental health outcomes among Central American and Mexican children held in immigration detention at the United States-Mexico border. Dev Psychobiol.

[31] Keller A, Joscelyne A, Granski M, Rosenfeld B (2017). Pre-Migration Trauma Exposure and Mental Health Functioning among Central American Migrants Arriving at the US Border. PLoS ONE.

[32] Chiesa V, Chiarenza A, Mosca D, Rechel B (2019). Health records for migrants and refugees: A systematic review. Health Policy.

