# Supplementary material for: Epidemiology of asylum seekers and refugees at the Mexico-US border: a cross-sectional analysis from the migrant settlement camp in Matamoros, Mexico
Source: BMC Public Health. 2024 Feb 16;24:489. doi: 10.1186/s12889-024-17947-7 (PMC10870647; doi:10.1186/s12889-024-17947-7)
Supplement: Supplementary file 1 — Additional file 1: Supplementary Material 1. Comprehensive list of ICD-10 diagnostic codes for people migrating in Matamoros, Mexico from November 2019 to March 2022. [file 12889_2024_17947_MOESM1_ESM.docx]

Supplementary Material 1: Comprehensive list of ICD-10 diagnostic codes for people migrating in Matamoros, Mexico from November 2019 to March 2022

| **ICD-10 classification** | **ICD-10 Sub-Chapter** | **Frequency (%)** |
| --- | --- | --- |
|  |  | (N=9744 diagnoses) |
| Symptoms, signs and abnormal clinical and laboratory findings, not elsewhere classified | | 4101 (42%) |
| Diseases of the respiratory system |  | 1466 (15%) |
|  | Acute Upper Respiratory Infections | 719 (7.4%) |
|  | Influenza And Pneumonia | 430 (4.4%) |
|  | Other Diseases Of Upper Respiratory Tract | 267 (2.7%) |
|  | Chronic Lower Respiratory Diseases | 43 (0.4%) |
|  | Other Acute Lower Respiratory Infections | 3 (0%) |
|  | Other Diseases Of The Respiratory System | 2 (0%) |
|  | Other Respiratory Diseases Principally Affecting the Interstitium | 1 (0%) |
| Diseases of the musculoskeletal system and connective tissue |  | 1081 (11.1%) |
|  | Other Dorsopathies | 440 (4.5%) |
|  | Other Soft Tissue Disorders | 340 (3.5%) |
|  | Other Joint Disorders | 262 (2.7%) |
|  | Disorders Of Bone Density And Structure | 13 (0.1%) |
|  | Disorders Of Muscles | 7 (0.1%) |
|  | Inflammatory Polyarthropathies | 5 (0.1%) |
|  | Osteoarthritis | 4 (0%) |
|  | Chondropathies | 3 (0%) |
|  | Systemic Connective Tissue Disorders | 2 (0%) |
|  | Deforming Dorsopathies | 1 (0%) |
|  | Dentofacial Anomalies [Including Malocclusion] And Other Disorders Of Jaw | 1 (0%) |
|  | Disorders Of Synovium And Tendon | 1 (0%) |
|  | Other Osteopathies | 1 (0%) |
| Diseases of the skin and subcutaneous tissue |  | 473 (4.8%) |
|  | Dermatitis And Eczema | 221 (2.3%) |
|  | Other Disorders Of The Skin And Subcutaneous Tissue | 120 (1.2%) |
|  | Disorders Of Skin Appendages | 60 (0.6%) |
|  | Infections Of The Skin And Subcutaneous Tissue | 42 (0.4%) |
|  | Urticaria And Erythema | 15 (0.2%) |
|  | Papulosquamous Disorders | 11 (0.1%) |
|  | Radiation-Related Disorders Of The Skin And Subcutaneous Tissue | 4 (0%) |
| Diseases of the genitourinary system |  | 451 (4.6%) |
|  | Noninflammatory Disorders Of Female Genital Tract | 306 (3.1%) |
|  | Disorders Of Breast | 49 (0.5%) |
|  | Inflammatory Diseases Of Female Pelvic Organs | 31 (0.3%) |
|  | Diseases Of Male Genital Organs | 20 (0.2%) |
|  | Other Diseases Of The Urinary System | 19 (0.2%) |
|  | Urolithiasis | 13 (0.1%) |
|  | Other Disorders Of Kidney And Ureter | 7 (0.1%) |
|  | Glomerular Diseases | 3 (0%) |
|  | Acute Kidney Failure And Chronic Kidney Disease | 1 (0%) |
|  | Renal Tubulo-Interstitial Diseases | 1 (0%) |
| Diseases of the digestive system |  | 435 (4.5%) |
|  | Diseases Of Oral Cavity And Salivary Glands | 270 (2.8%) |
|  | Other Diseases Of Intestines | 68 (0.7%) |
|  | Diseases Of Esophagus, Stomach And Duodenum | 65 (0.7%) |
|  | Hernia | 10 (0.1%) |
|  | Other Diseases Of The Digestive System | 8 (0.1%) |
|  | Disorders Of Gallbladder, Biliary Tract And Pancreas | 7 (0.1%) |
|  | Noninfective Enteritis And Colitis | 4 (0%) |
|  | Diseases Of Liver | 3 (0%) |
| Injury, poisoning and certain other consequences of external causes |  | 346 (3.5%) |
|  | Injuries To The Head | 75 (0.8%) |
|  | Injuries To The Ankle And Foot | 49 (0.5%) |
|  | Other And Unspecified Effects Of External Causes | 39 (0.4%) |
|  | Injuries To The Knee And Lower Leg | 34 (0.3%) |
|  | Injuries To The Wrist, Hand And Fingers | 30 (0.3%) |
|  | Burns And Corrosions Of External Body Surface, Specified By Site | 22 (0.2%) |
|  | Toxic Effects Of Substances Chiefly Nonmedicinal As To Source | 20 (0.2%) |
|  | Injuries To The Elbow And Forearm | 16 (0.2%) |
|  | Effects Of Foreign Body Entering Through Natural Orifice | 11 (0.1%) |
|  | Injuries To The Shoulder And Upper Arm | 8 (0.1%) |
|  | Injuries To The Abdomen, Lower Back, Lumbar Spine, Pelvis And External Genitals | 7 (0.1%) |
|  | Poisoning By, Adverse Effects Of And Underdosing Of Drugs, Medicaments And Biological Substances | 7 (0.1%) |
|  | Injuries To The Thorax | 6 (0.1%) |
|  | Injury Of Unspecified Body Region | 6 (0.1%) |
|  | Injuries To The Neck | 5 (0.1%) |
|  | Complications Of Surgical And Medical Care, Not Elsewhere Classified | 3 (0%) |
|  | Injuries To The Hip And Thigh | 2 (0%) |
|  | Injuries Involving Multiple Body Regions | 2 (0%) |
|  | Burns And Corrosions Of Multiple And Unspecified Body Regions | 1 (0%) |
|  | Certain Early Complications Of Trauma | 1 (0%) |
| Diseases of the eye and adnexa |  | 311 (3.2%) |
|  | Other Disorders Of Eye And Adnexa | 179 (1.8%) |
|  | Disorders Of Eyelid, Lacrimal System And Orbit | 50 (0.5%) |
|  | Visual Disturbances And Blindness | 39 (0.4%) |
|  | Disorders Of Conjunctiva | 33 (0.3%) |
|  | Disorders Of Vitreous Body And Globe | 5 (0.1%) |
|  | Disorders Of Ocular Muscles, Binocular Movement, Accommodation And Refraction | 4 (0%) |
|  | Disorders Of Sclera, Cornea, Iris And Ciliary Body | 1 (0%) |
| Diseases of the ear and mastoid process |  | 293 (3%) |
|  | Other Disorders Of Ear | 264 (2.7%) |
|  | Diseases Of External Ear | 13 (0.1%) |
|  | Diseases Of Middle Ear And Mastoid | 11 (0.1%) |
|  | Diseases Of Inner Ear | 4 (0%) |
| Certain infectious and parasitic diseases |  | 176 (1.8%) |
|  | Mycoses | 78 (0.8%) |
|  | Helminthiases | 32 (0.3%) |
|  | Viral Infections Characterized By Skin And Mucous Membrane Lesions | 27 (0.3%) |
|  | Pediculosis, Acariasis And Other Infestations | 20 (0.2%) |
|  | Infections With A Predominantly Sexual Mode Of Transmission | 6 (0.1%) |
|  | Other Viral Diseases | 4 (0%) |
|  | Human Immunodeficiency Virus [HIV] Disease | 2 (0%) |
|  | Intestinal Infectious Diseases | 2 (0%) |
|  | Other Bacterial Diseases | 2 (0%) |
|  | Protozoal Diseases | 2 (0%) |
|  | Bacterial And Viral Infectious Agents | 1 (0%) |
| Endocrine, nutritional and metabolic diseases |  | 144 (1.5%) |
|  | Other Nutritional Deficiencies | 61 (0.6%) |
|  | Diabetes Mellitus | 49 (0.5%) |
|  | Disorders Of Thyroid Gland | 15 (0.2%) |
|  | Metabolic Disorders | 13 (0.1%) |
|  | Disorders Of Other Endocrine Glands | 4 (0%) |
|  | Malnutrition | 2 (0%) |
| Diseases of the nervous system |  | 126 (1.3%) |
|  | Episodic And Paroxysmal Disorders | 103 (1.1%) |
|  | Other Disorders Of The Nervous System | 14 (0.1%) |
|  | Nerve, Nerve Root And Plexus Disorders | 5 (0.1%) |
|  | Cerebral Palsy And Other Paralytic Syndromes | 2 (0%) |
|  | Extrapyramidal And Movement Disorders | 1 (0%) |
|  | Polyneuropathies And Other Disorders Of The Peripheral Nervous System | 1 (0%) |
| External causes of morbidity |  | 97 (1%) |
|  | Slipping, Tripping, Stumbling And Falls | 38 (0.4%) |
|  | Exposure To Inanimate Mechanical Forces | 21 (0.2%) |
|  | Exposure To Animate Mechanical Forces | 16 (0.2%) |
|  | Accidental Exposure To Other Specified Factors | 4 (0%) |
|  | Supplementary Factors Related To Causes Of Morbidity Classified Elsewhere | 4 (0%) |
|  | Contact With Heat And Hot Substances | 2 (0%) |
|  | Assault | 1 (0%) |
|  | Event Of Undetermined Intent | 1 (0%) |
|  | Exposure To Smoke, Fire And Flames | 1 (0%) |
|  | Other Land Transport Accidents | 1 (0%) |
|  | Overexertion And Strenuous Or Repetitive Movements | 1 (0%) |
|  | Pedal Cycle Rider Injured In Transport Accident | 1 (0%) |
| Diseases of the circulatory system |  | 86 (0.9%) |
|  | Hypertensive Diseases | 70 (0.7%) |
|  | Pulmonary Heart Disease And Diseases Of Pulmonary Circulation | 6 (0.1%) |
|  | Other Forms Of Heart Disease | 5 (0.1%) |
|  | Ischemic Heart Diseases | 2 (0%) |
|  | Cerebrovascular Diseases | 1 (0%) |
|  | Diseases Of Arteries, Arterioles And Capillaries | 1 (0%) |
|  | Other And Unspecified Disorders Of The Circulatory System | 1 (0%) |
| Pregnancy, childbirth and the puerperium |  | 85 (0.9%) |
|  | Other Maternal Disorders Predominantly Related To Pregnancy | 46 (0.5%) |
|  | Other Obstetric Conditions, Not Elsewhere Classified | 15 (0.2%) |
|  | Pregnancy With Abortive Outcome | 14 (0.1%) |
|  | Maternal Care Related To The Fetus And Amniotic Cavity And Possible Delivery Problems | 4 (0%) |
|  | Supervision Of High Risk Pregnancy | 3 (0%) |
|  | Complications Predominantly Related To The Puerperium | 2 (0%) |
|  | Edema, Proteinuria And Hypertensive Disorders In Pregnancy, Childbirth And The Puerperium | 1 (0%) |
| Mental, Behavioral and Neurodevelopmental disorders |  | 45 (0.5%) |
| Diseases of the blood and blood-forming organs and certain disorders involving the immune mechanism |  | 21 (0.2%) |
| Neoplasms |  | 14 (0.1%) |
| Provisional assignment of new diseases of uncertain etiology or emergency use |  | 12 (0.1%) |
| Congenital malformations, deformations and chromosomal abnormalities |  | 11 (0.1%) |
