# Supplementary material for: Epidemiology of asylum seekers and refugees at the Mexico-US border: a cross-sectional analysis from the migrant settlement camp in Matamoros, Mexico
Source: BMC Public Health. 2024 Feb 16;24:489. doi: 10.1186/s12889-024-17947-7 (PMC10870647; doi:10.1186/s12889-024-17947-7)
Supplement: Supplementary file 2 — Additional file 2: Supplemental Material 2. Complete list of medications distributed to migrating people in Matamoros, Mexico, categorized according to the World Health Organization List of Essential Medicines. [file 12889_2024_17947_MOESM2_ESM.docx]

Supplemental Material 2: Complete list of medications distributed to migrating people in Matamoros, Mexico, categorized according to the World Health Organization List of Essential Medicines

| **Medication category** | **Medication** | **Frequency (n=7145)** | **Percentage (%)** |
| --- | --- | --- | --- |
| Medicines for Pain and Palliative Care |  | 2181 | 30.5 |
|  | NSAID | 1028 | 14.4 |
|  | Analgesic + Antipyretic | 780 | 10.9 |
|  | NSAID + Supplement | 136 | 1.9 |
|  | Antispasmodic | 83 | 1.2 |
|  | NSAID + Anticoagulant | 40 | 0.6 |
|  | Anesthetic | 37 | 0.5 |
|  | Opioid | 27 | 0.4 |
|  | Antispastic | 14 | 0.2 |
|  | Anesthetic + Steroid + Skin Protectant | 12 | 0.2 |
|  | Analgesic | 9 | 0.1 |
|  | Anti-inflammatory | 7 | 0.1 |
|  | CCB + Antispasmodic | 4 | 0.1 |
|  | NSAID + Anesthetic | 3 | <0.1 |
|  | NSAID + Narcotic | 1 | <0.1 |
| Anti-Infective Medicines |  | 1611 | 22.5 |
|  | Antibiotic | 853 | 11.9 |
|  | Antifungal | 423 | 5.9 |
|  | Antiparasitic | 213 | 3.0 |
|  | Antibiotic + Steroid + Anesthetic | 46 | 0.6 |
|  | Antibiotic Ointment | 29 | 0.4 |
|  | Antibiotic + Antifungal | 26 | 0.4 |
|  | Antiviral + Antihistamine + Analgesic + Antipyretic | 6 | 0.1 |
|  | Antiviral | 4 | 0.1 |
|  | Antibiotic + Antidiarrheal | 3 | <0.1 |
|  | Albendazole | 2 | <0.1 |
|  | Antibiotic + Analgesic | 2 | <0.1 |
|  | Antibiotic + Steroid | 2 | <0.1 |
|  | Antiviral + Antihistamine + Analgesic | 1 | <0.1 |
|  | Silver Sulfadiazine | 1 | <0.1 |
| Ear, Nose and Throat Medicines |  | 1021 | 14.3 |
|  | Decongestant + Cough Suppression | 338 | 4.7 |
|  | Analgesic + Antipyretic + Muscle Relaxant | 233 | 3.3 |
|  | Analgesic + Antipyretic + Decongestant + Antihistamine | 178 | 2.5 |
|  | Decongestant | 116 | 1.6 |
|  | Cough Suppressant + Analgesic | 73 | 1.0 |
|  | Analgesic + Antipyretic + Decongestant + Cough Suppressant | 42 | 0.6 |
|  | Cold Medication | 14 | 0.2 |
|  | Decongestant + Vasoconstrictor | 14 | 0.2 |
|  | Analgesic + Antipyretic + Decongestant | 4 | 0.1 |
|  | Decongestant + Moisturizer | 4 | 0.1 |
|  | Cough Medicine | 3 | <0.1 |
|  | Nasal | 2 | <0.1 |
| Antiallergics and Medicines Used in Anaphylaxis |  | 605 | 8.5 |
|  | Antihistamine | 557 | 7.8 |
|  | Antihistamine + Antacid | 42 | 0.6 |
|  | Allergy (unspecified) | 6 | 0.1 |
| Vitamins and Minerals |  | 601 | 8.4 |
|  | Supplement | 601 | 8.4 |
| Medicines for Endocrine Disorders |  | 443 | 6.2 |
|  | Steroid | 279 | 3.9 |
|  | Hormonal | 92 | 1.3 |
|  | Diabetes Management | 69 | 1.0 |
|  | SERM | 2 | <0.1 |
|  | Hormone | 1 | <0.1 |
| Gastrointestinal Medicines |  | 296 | 4.1 |
|  | Antacid | 127 | 1.8 |
|  | PPI | 95 | 1.3 |
|  | Anti-emetic | 37 | 0.5 |
|  | Antidiarrheal | 13 | 0.2 |
|  | Laxative | 8 | 0.1 |
|  | Probiotics | 8 | 0.1 |
|  | Ulcer Protectant | 3 | <0.1 |
|  | Anti-bloat | 2 | <0.1 |
|  | Gallstone Dissolution | 2 | <0.1 |
|  | Ondansetron | 1 | <0.1 |
| Solutions Correcting Water, Electrolyte and Acid-Base Disturbances |  | 119 | 1.7 |
|  | Electrolytes | 119 | 1.7 |
| Cardiovascular Medicines |  | 117 | 1.6 |
|  | Antihypertensive | 116 | 1.6 |
|  | Anticoagulant | 1 | <0.1 |
| Dermatological Medicines (Topical) |  | 105 | 1.5 |
|  | Steroid + Antibiotic + Skin Protectant | 60 | 0.8 |
|  | Skin Protectant | 23 | 0.3 |
|  | Rash Barrier | 22 | 0.3 |
| Medicines for Reproductive Health and Perinatal Care |  | 15 | 0.2 |
|  | Contraction Inducer | 14 | 0.2 |
|  | Expectorant | 1 | <0.1 |
| Ophthalmological Preparations |  | 14 | 0.2 |
|  | Eye Drops | 14 | 0.2 |
| Anticonvulsants/Antiepileptics |  | 10 | 0.1 |
|  | Anti-epileptic | 8 | 0.1 |
|  | Anti-convulsant | 2 | <0.1 |
| Medicines for Mental and Behavioural Disorders |  | 3 | <0.1 |
|  | Benzodiazepine | 1 | <0.1 |
|  | Paroxetine | 1 | <0.1 |
|  | SSRI | 1 | <0.1 |
| Diuretics |  | 2 | <0.1 |
|  | Diuretic | 2 | <0.1 |
| Antimigraine Medicines |  | 1 | <0.1 |
|  | Antimigraine | 1 | <0.1 |
| Immunomodulators and Antineoplastics |  | 1 | <0.1 |
|  | Chemotherapy | 1 | <0.1 |
| TOTAL |  | 7145 | 100 |
